# Fewer actionable mutations but higher tumor mutational burden characterizes NSCLC in black patients at an urban academic medical center

**DOI:** 10.18632/oncotarget.27212

**Published:** 2019-10-08

**Authors:** Noura J. Choudhury, Mansooreh Eghtesad, Sabah Kadri, John Cursio, Lauren Ritterhouse, Jeremy Segal, Aliya Husain, Jyoti D. Patel

**Affiliations:** ^1^ Department of Medicine, The University of Chicago, Chicago, Illinois, USA; ^2^ Department of Pathology, Advocate Illinois Masonic Medical Center, Chicago, Illinois, USA; ^3^ Department of Pathology, Ann and Robert H. Lurie Children's Hospital of Chicago, Chicago, Illinois, USA; ^4^ Department of Public Health Sciences, The University of Chicago, Chicago, Illinois, USA; ^5^ Division of Genomic and Molecular Pathology, Department of Pathology, The University of Chicago, Chicago, Illinois, USA; ^6^ Department of Pathology, The University of Chicago, Chicago, Illinois, USA

**Keywords:** non-small cell lung cancer, healthcare disparities, targeted therapies, immunotherapies, biomarkers

## Abstract

**Background:** Black patients have been historically underrepresented in studies investigating molecular patterns in non-small cell lung cancer (NSCLC). We aimed to investigate differences in actionable mutations among patients at our urban, diverse medical center.

**Results:** 146 patients were included (59 black, 76 white, 7 Asian, 3 Hispanic, 1 mixed). 35 patients had a targetable mutation. Seven black patients (11.8%) had a targetable mutation compared to 28 non-black patients (32.2%, *p* = 0.005). 15 black patients had PD-L1 expression ≥50% compared to 19 non-black (25.4% vs 21.8%, *p* = 0.69). Black patients had a higher TMB compared to non-black (15.3 mutations/Mb compared to 11.5 mutations/Mb, *p* = 0.001). In a multivariate analysis, TMB was driven by smoking (*p* < 0.01), without any additive interaction in black patients who smoke (*p* = 0.8).

**Conclusion:** NSCLC tumors from black patients had a higher TMB and were less likely to carry a targetable mutation. The higher TMB seen was driven by a higher prevalence of smoking among black patients in our study, which may not reflect nationwide trends. Our results serve as a proof of concept that differences in molecular markers exist between black and non-black patients, and that these differences may impact the treatment options available to black patients.

**Methods:** Retrospective chart review of patients with a diagnosis of NSCLC who underwent both PD-L1 testing and massively parallel sequencing (UCM-OncoPlus) was conducted. We examined whether high PD-L1 expression, tumor mutational burden (TMB), and presence of targetable mutations (*EGFR*, *BRAF*, *ERBB2*, *RET* or *ALK* translocations, *ROS1* rearrangements) occur at different frequencies in tumors from black patients compared to non-black patients.

## INTRODUCTION

Black male patients with lung cancer are 1.2 times more likely to die from their disease compared to white male patients, a modest improvement from 1.4 times in the 1990s [1]. In others cancer, a higher mortality rate can be partially explained by factors such as a higher incidence of more aggressive disease among black patients. For example, women with African ancestry are known to develop triple-negative breast cancer at higher rates [2]. A similar link has not been found for lung cancer. While investigation into the molecular composition of lung cancer among black patients has been limited, it continues to be essential to address mortality disparities.

Best practice for the treatment of advanced, non-squamous non-small cell lung cancer (NSCLC) includes identification of targetable or actionable mutations such as in *EGFR*, *ALK*, *ROS1* and *BRAF* to guide treatment selection [3]. Programmed death-ligand 1 (PD-L1) assessment is also broadly recommended, as single agent pembrolizumab can be offered as first-line therapy in patients whose tumors express high levels of PD-L1. More recently, high tumor mutational burden (TMB) has been associated with treatment response to immunotherapies in lung cancer [4]. Despite this, molecular testing remains underutilized, with reduced uptake among minorities such as black and Hispanic patients [5, 6]. While several studies have investigated the frequencies of targetable mutations in black patients with NSCLC, these studies have yielded conflicting results [7] and have not included PD-L1, TMB and actionable mutations comprehensively. It therefore remains unclear whether targeted therapies and immunotherapies disproportionately benefit non-black patients, both because of disparities in access to molecular testing as well as potentially higher prevalence of actionable mutations among non-black patients.

We sought to investigate whether differences in the molecular composition of NSCLC among our diverse patient population at an urban academic medical center impact the treatment options available for underserved patients. Since early 2016, all patients with a diagnosis of NSCLC at our institution underwent both targeted sequencing with the UCM-OncoPlus panel [7], as well as PD-L1 immunohistochemistry (IHC), even if the initial cancer diagnosis was made in the inpatient setting, or if patients transferred their care from another center. As an academic, tertiary care medical center located on the south side of Chicago, we are able to offer routine molecular testing that otherwise may not be available to underserved patients in the area. Of note, we focus in this study on “actionable” or “targetable” mutations, which we use to denote molecular alterations which are currently the targets of commercially-available drugs approved for use in NSCLC. In addition, while there is significant heterogeneity in the academic or scientific literature in the terms used to describe race or ethnicity [8], we employ the categories “black,” “white”, “Asian” and “Hispanic or Latino,” as set forth by the National Institutes of Health to describe self-reported race [9].

## RESULTS

146 patients were included as follows: 59 (40.4%) black patients, 76 (52.1%) white, 7 Asians (4.8%), 3 Hispanic (2.1%), and one patient of mixed race. Patient characteristics are outlined in Table 1. The majority of patients were stage IV at the time of molecular testing (91 patients, 62.3%). 27 (25.3%) patients were light or never smokers compared to 96 (65.7%) heavy former or current smokers. A higher prevalence of any smoking history was noted among black patients, with 48/53 (90.6%) black patients reporting a smoking history versus 53/87 (60.9%) non-black patients reporting a smoking history (*p* = 0.003).

**Table 1:** Patient characteristics

Characteristic	Black	Non-black	Total number (%)
Gender			
Male	22	44	66 (45.2)
Female	37	43	80 (54.8)
Median age at diagnosis	66	67	66 years
Stage			
I or II	5	15	20 (13.7)
III	6	24	30 (20.5)
IV	38	53	91 (62.3)
Not sufficiently reported	4	1	5 (3.4)
Histology			
Adenocarcinoma	49	69	118 (80.8)
Squamous cell carcinoma	2	8	10 (6.8)
Neuroendocrine	1	1	2 (1.4)
Rare or mixed features	4	2	6 (4.1)
Not further differentiated	3	7	10 (6.8)
Smoking History			
Never smoker	2	23	25 (17.1)
Light former smoker (<10 py)	7	5	12 (8.2)
Heavy former smoker (≥10 py)	33	47	80 (54.8)
Current smoker	11	5	16 (10.9)
Not sufficiently reported	6	7	13 (8.9)
Received a TKI	4	16	20 (13.7)
Received an immunotherapy	16	26	42 (28.7)
Self-reported race			
Black			59 (40.4)
White			76 (52.1)
Asian			7 (4.8)
Hispanic/Latino			3 (2.1)
Mixed			1 (0.7)

TKI=“tyrosine kinase inhibitor,” py= pack-years. 146 patients were included in the final analysis. “Not sufficiently reported” indicates electronic medical records do not provide enough information to place patients in the assigned categories. Stage was determined by stage reported at the time of molecular testing. Under “Histology,” “mixed or rare features” includes tumors that were characterized as adenosquamous, rhabdoid, sarcomatoid, and one patient with mixed small cell and adenocarcinoma. Of note, nine patients transferred their care after undergoing molecular work-up at UCMC and their clinical course and treatments are not known.

35 patients had at least one targetable mutation, with *EGFR* alterations seen in 21 patients, 16 of whom were white, as shown in Table 2. Two white patients had both an *EGFR* mutation and a *CCDC6-RET* fusion. Seven black patients had a targetable mutation (11.9%) compared to 24 (31.5%) white patients and 28 total non-black patients (*p* = 0.005, Fisher’s exact). The presence of a targetable alteration was strongly associated with light or never smoking (*p <* 0.0001).

**Table 2:** Summary of molecular alterations

A	Black	White	Asian	Hispanic/Other	Total
**Targetable Alterations**					No. (%)
*EGFR*	4	16	1	0	21 (14.4)
*BRAF*	0	0	0	1	1 (0.1)
*ERBB2*	1	3	0	0	4 (2.7)
*MET* exon 14	2	3	1	0	6 (4.1)
*RET* translocation	0	3	0	0	3 (2.1)
*ALK* rearrangement	0	0	1	0	1 (0.1)
*ROS1* rearrangement	0	1	0	0	1 (0.1)
No. (%)	7 (11.9)	26 (34.2)	3 (42.9)	1 (20.0)	
**PD-L1 expression**					
<1%	35	37	1	4	77 (52.7)
≥1 to 49%	9	24	2	0	35 (24.0)
≥50%	15	15	4	0	34 (23.2)
**TMB (mutations/Mb)**	15.3 ± 11.2	12.3 ± 16.1	6.5 ± 3.0	6.9 ± 6.2	*p*-value 0.006
Mean ± SD	*n* = 53	*n* = 66	*n* = 7	*n* = 4	
**B**	**Black**	**Non-black**	***p*-value **		
Targetable Mutations	7 (11.9)	28 (32.2)	0.005		
High PD-L1 expression (≥50%)	15 (25.4)	19 (21.8)	0.69		
TMB (mean ± SD)	15.3 ± 11.2	11.5 ± 15.1	0.001		

In Table 2A, the distribution of targetable mutations and PD-L1 expression among the four racial groups is shown above. Two white patients had both an *EGFR* activating mutation as well as a *CCDC6*-*RET* fusion, resulting in a total of 35 unique patients possessing at least one targetable mutation. The 3 Hispanic/Latino patients and the one patient of mixed race were grouped together. 130 patients underwent tumor mutation burden (TMB) analysis, with sample size per race listed above. *p*-value for TMB distribution among the four groups was determined using Kruskal-Wallis test given non-parametric distribution. In Table 2B, patients are distinguished as black vs. white, Asian, Hispanic and mixed.

PD-L1 expression was scored as either low (<1%), medium (≥1 to 49%), or high (≥50%) tumor cell expression. Overall, 77 (52.7%) patient tumors had low expression, 35 (24.0%) medium, and 34 (23.2%) high expression. There was no difference in high PD-L1 expression between black patients and non-black patients (*p* = 0.69, Fisher’s exact). This remained non-significant when the threshold for high PD-L1 expression was lowered to ≥1% (*p* = 0.23).

**Figure 1:**
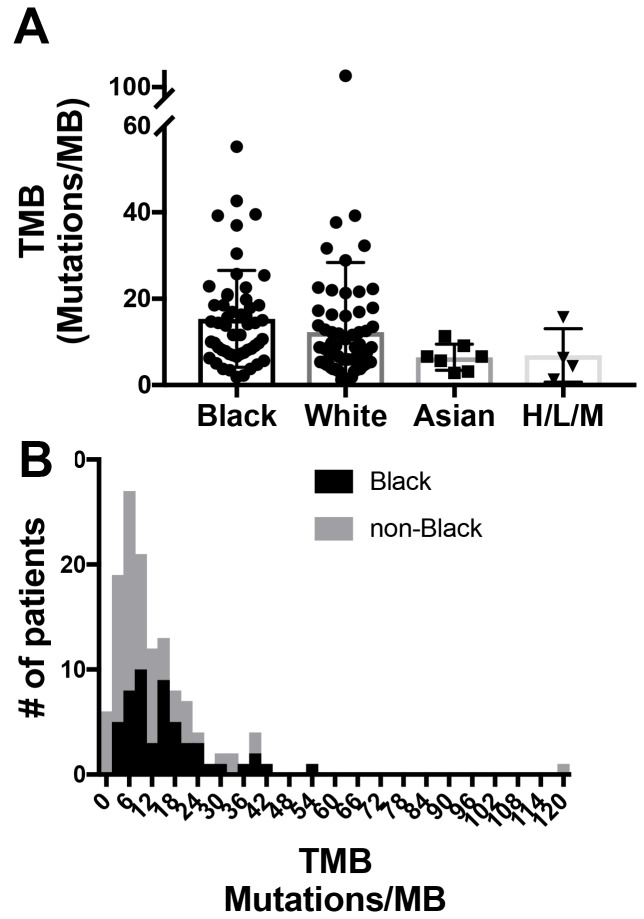
Distribution of tumor mutational burden. (**A**) demonstrates the distribution of tumor mutation burden (TMB) (mutations/ megabase or MB) for each racial group. Sample size for each group is noted on the X-axis. *p* = 0.006 (Kruskal-Wallis). H/M designates “Hispanic/mixed”. These categories were combined due to small numbers. (**B**) is a histogram of the distribution of TMB for black vs. non-black patients, with TMB on the X-axis. *p* = 0.001 (Mann-Whitney *U* test).

Finally, the average TMB was calculated for each group (Table 2). There was a significant difference in TMB among the four racial groups (*p* = 0.006,). Black patients had the highest TMB, with a mean of 15.3 mutations/Mb, compared to a mean of 11.5 mutations/Mb for non-black patients (*p* = 0.001, Mann-Whitney *U* test) (Figure 1). When stratified according to smoking status, we found that the results were no longer significant. In a multivariable regression model, we found that smoking, rather than black race, was significantly associated with a higher mean TMB (*p* = 0.003) with a non-significant interaction between black race and smoking (*p* = 0.8 for the interaction coefficient).

Given that this study included patients with all stages of disease, we sought to ascertain whether the proportion of tumors with targetable mutations or higher TMB was potentially associated with stage. We found no difference between average TMB between metastatic and non-metastatic disease groups (11.3 vs. 16.3, *p* = 0.25 by Mann-Whitney U), or in the proportion of tumors with a targetable mutation. 8/50 (16.0%) patients with non-metastatic disease had a targetable mutation, compared to 27/91 (29.6%) patients with metastatic disease (*p* = 0.07).

## DISCUSSION

Our study incorporates PD-L1 testing, TMB quantification and clinically actionable mutation analysis together, which best reflects the comprehensive molecular testing recommended to guide treatment decisions. With fewer actionable mutations, the implications of our study are that black patients have less opportunity to benefit from targeted therapies that are commercially available, which significantly extend survival for eligible patients. Black and non-black patients, conversely, may be similarly eligible for immunotherapies. In fact, only 4/59 (6.8%) black patients among our patients received a tyrosine-kinase inhibitor among our patients, compared to 14 (18.4%) white patients in the cohort. In comparison, nearly equal proportions received immunotherapies (27% of black patients and 25% of white patients). Of note, some patients with actionable mutations or high PD-L1 expression may not have received a targeted therapy or immunotherapy if they presented with localized disease as this was not the standard of care at the time. Furthermore, fewer patients in our cohort at the time of data collection received an immunotherapy than would today, since the period of data collection preceded expanded U. S. Food and Drug Administration (FDA) approval for the use of immunotherapies in NSCLC. The expanded indications for using immunotherapies in NSCLC available today are likely to benefit both black and non-black patients.

Characterizing the frequencies of targetable mutations in NSCLC tumors from black patients has been of significant interest, with *EGFR* the most commonly investigated. Several studies report a lower frequency of *EGFR* mutations in tumors from black patients [10–12], even after correcting for smoking status [13], while others did not show a difference [14–16]. Compared to several of these studies, our study directly compares patients from different racial groups using synchronous testing from a single institution, which reduces potential sources of error. Similar methodology was employed in a 2017 study by Campbell, *et al.* [17].

The molecular differences in our study population appear to be driven by a higher prevalence of smoking in our urban black population compared to other groups. In fact, the discrepancy in mutation frequency reported among published studies are likely due to variable proportions of smokers in each study [7]. Smoking prevalence will undoubtedly vary across institutions and regions and the patterns of smoking in our population do not necessarily reflect nationwide trends.

Our study has several limitations, including smaller sample size, especially of Asian and Hispanic patients. Because of this, our study is not meant to conclusively demonstrate differences in molecular composition among patients. In addition, the retrospective nature of our study curtailed the ability to precisely correlate molecular alterations to treatment response, especially as patients may have received combination or sequential therapies. However, even with a smaller sample size, differences in molecular alterations between black and non-black patients were still elucidated. Furthermore, our sample confirmed several trends that have been demonstrated by multiple larger studies and which are embraced in the literature, including that higher TMB is associated with smoking [18], and that targetable mutations are found more commonly in non-smokers [19]. These findings offer assurance of the validity of our sample, despite its smaller size.

## METHODS

The University of Chicago Institutional Review Board granted an exemption for this retrospective study. All patients of any tumor stage at any point of their treatment with a pathological diagnosis of NSCLC who had a UCM-OncoPlus performed from March 2016 to October 2017 were screened. Patients were included if they had PD-L1 IHC as well as UCM-OncoPlus results. Electronic medical records (EMR) were reviewed with clinical data extracted and identifying personal information removed. Smoking history was characterized by current vs. former use and heavy (smoking history ≥ 10 pack-years) vs. light use. Of note, race was self-reported by patients. “Hispanic or Latino” is listed as a category for race in our EMR. Patients can choose to further specify ethnicity as a separate category, but fewer do so. Stage was also determined as the stage denoted in the EMR at the time molecular testing was performed.

Targeted next-generation sequencing was ordered by clinicians directly caring for patients. This is performed on tumor samples using the University of Chicago’s validated panel, the UCM-OncoPlus, which covers 1,213 cancer-related genes [20]. We defined “targetable” mutations as druggable alterations in *EGFR* (exon 18–21 alterations), *BRAF* (V600E), *ERBB2, MET* exon 14 skip mutations, *RET* translocations, and *ALK* and *ROS1* rearrangements that are sensitizing to FDA-approved agents. TMB was quantified as mutations/Mb using UCM-OncoPlus results. Briefly, variants were restricted to genes with >10% variant allele frequency. Inherited SNPs were removed using 1000 Genome and EXAC databases. Genes with pseudogene noise and sequencing artifacts were removed from the analysis. Finally, a COSMIC rescue was performed using a >10 COSMIC entry threshold on variants with lower than 0.1% ExAC frequency. Tumor samples also underwent IHC with the Dako PD-L1 clone 28.8 antibody (Agilent, Santa Clara, CA, USA), with expression quantified by two pathologists (ME and AH).

Statistical analysis was carried out by a biostatistician (JC) using SAS software, version 9.4. All *p*-values are two-sided with significance cut off of ≤0.05.

## CONCLUSIONS

Our study demonstrates a lower frequency of actionable mutations but a higher TMB among black patients in our urban, racially diverse population. The driving factors behind this pattern is likely the higher prevalence of smoking among black patients compared to non-black patients in our population. Our results do not suggest that black patients should be profiled as smokers or empirically offered immunotherapies. Due to our smaller sample size, our results are also not intended to be used as a definitive synopsis of the difference in molecular alterations between black and non-black patients. Instead, our study instead serves as a proof of concept that differences in molecular alterations between black and non-black patients with NSCLC exist and do impact the opportunities of patients to receive novel therapies. These results also demonstrate the necessity of improving access to guideline-directed molecular testing for all patients, especially those who may not have had access to such testing in underserved communities. Addressing sociodemographic inequalities, including access to guideline-directed molecular testing, inclusion in biomarker-discovery and therapeutic clinical trials, and continued investigation into the molecular analysis of NSCLC in black patients, is essential to reducing the excess mortality from NSCLC that black patients experience.

## Abbreviations

PD-L1: Programmed death-ligand 1
NSCLC: non-small cell lung cancer
TMB: tumor mutational burden
IHC: immuno-histochemistry
EMR: electronic medical records
FDA: U.S. Food and Drug Administration
